# Surgical management of endometriosis: surgical techniques, adjuvant therapy, and long-term management Number 10 – 2026

**DOI:** 10.61622/rbgo/2026FPS10

**Published:** 2026-07-15

**Authors:** Agnaldo Lopes da Silva, Sérgio Podgaec, Ricardo de Almeida Quintarios, Júlio César Rosa e Silva

**Affiliations:** 1 Universidade Federal de Minas Gerais Belo Horizonte MG Brazil Universidade Federal de Minas Gerais, Belo Horizonte, MG, Brazil; 2 Universidade de São Paulo São Paulo SP Brasil Universidade de São Paulo, São Paulo, SP, Brasil; 3 Hospital Israelita Albert Einstein São Paulo SP Brazil Hospital Israelita Albert Einstein, São Paulo, SP, Brazil; 4 Universidade do Estado do Pará Belém PA Brazil Universidade do Estado do Pará, Belém, PA, Brazil; 5 Faculdade de Medicina Universidade de São Paulo Ribeirão Preto SP Brazil Faculdade de Medicina, Universidade de São Paulo, Ribeirão Preto, SP, Brazil

## Abstract

• Surgical outcomes in endometriosis vary substantially according to disease phenotype, symptom profile, and reproductive priorities.

• Excisional surgery for ovarian endometriomas is associated with lower recurrence rates but carries a consistent risk of postoperative ovarian reserve impairment.

• Surgery for deep infiltrating endometriosis is associated with meaningful improvement in pain and quality of life when clinical indications are appropriate and execution is precise, but entails higher surgical complexity and morbidity.

• Repeat surgical interventions are associated with diminishing symptomatic benefit and increasing cumulative risk, including adhesion-related morbidity and the loss of both organ function and ovarian reserve.

• Postoperative hormonal therapy may reduce symptom recurrence and the likelihood of repeat surgery in women not seeking immediate pregnancy.

• Endometriosis surgery should be integrated into a long-term, multidisciplinary care model focused on symptom control and quality of life rather than disease eradication.

## Recommendations

- Surgical technique should be selected according to disease phenotype, symptom severity, and reproductive goals.
- For superficial peritoneal disease, excision or ablation may be considered in selected symptomatic women, while conservative management remains appropriate for mild disease.
- For ovarian endometriomas, laparoscopic cystectomy by excision should be preferred when surgery is indicated, using techniques that minimize damage to ovarian tissue and avoid excessive thermal injury.
- Conservative or ablative approaches may be considered in selected women with diminished ovarian reserve or high fertility priority, acknowledging the higher risk of recurrence.
- Surgical treatment of deep infiltrating endometriosis should be performed in specialized centers with multidisciplinary expertise and reserved for women with significant symptoms or documented organ dysfunction.
- Postoperative hormonal therapy should be offered to most women not seeking immediate pregnancy to reduce recurrence and symptom relapse.
- Repeat surgery should be avoided whenever possible and reserved for clearly defined indications after failure of optimized medical and multidisciplinary management.

## Background

**Figure 1:**
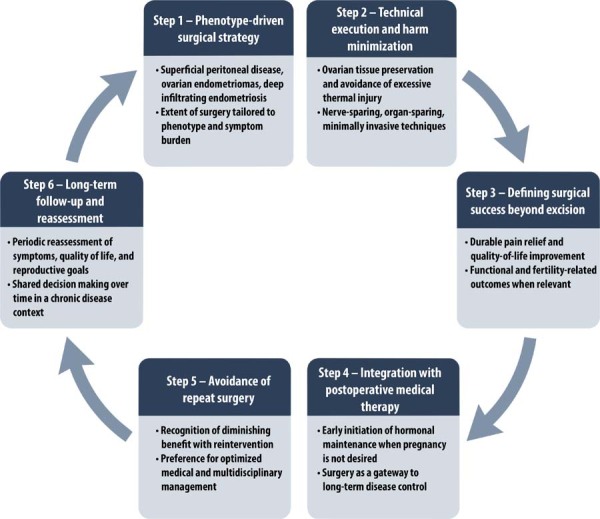
Framework for delivering long-term value after endometriosis surgery

When surgery is indicated, the surgical approach and perioperative strategy should be tailored to disease phenotype and patient goals. Minimally invasive surgery is the preferred approach for most women. In superficial peritoneal disease, both excision and ablation may be considered, as available evidence does not demonstrate clear superiority of one technique over the other. For ovarian endometriomas, laparoscopic cystectomy by excision is associated with lower rates of recurrence and higher spontaneous pregnancy rates, but carries a relevant risk of ovarian reserve reduction, requiring careful patient selection and preoperative counseling.^(1-4)^Surgical excision is recommended for deep infiltrating endometriosis, particularly when associated with organ dysfunction or severe, refractory symptoms. These procedures are technically complex and should be performed in specialized centers with multidisciplinary expertise to reduce morbidity and optimize outcomes.^(1,3-5)^ Radical surgery should be reserved for highly selected cases in women without future fertility plans.Postoperative medical therapy is a central component of long-term management. Continuous hormonal suppression with combined oral contraceptives, progestins, or levonorgestrel-releasing intrauterine systems may reduce symptom recurrence and disease relapse in women not seeking pregnancy.^(3-7)^ Discontinuation of therapy is associated with rapid loss of benefit and increased recurrence risk. Long-term follow-up should reassess symptoms, quality of life, and reproductive plans, recognizing endometriosis as a lifelong condition with fluctuating needs. Repeat surgery should be avoided whenever possible, given the cumulative risks and diminishing benefit over time.^(1,2,6)^This Position Statement supports phenotype-specific surgical strategies integrated with postoperative hormonal maintenance and individualized long-term follow-up, aiming to optimize outcomes while minimizing unnecessary interventions. Figure 1 summarizes a longitudinal framework integrating surgical technique, postoperative medical therapy, and follow-up to optimize long-term outcomes after endometriosis surgery.

### Which surgical techniques are recommended according to different endometriosis phenotypes?

Endometriosis presents with distinct phenotypes, including superficial peritoneal disease, ovarian endometriomas, and deep infiltrating endometriosis. Surgical treatment is indicated for refractory pain, organ involvement, infertility in selected cases, or suspicion of malignancy, and must be individualized according to symptom burden, impact on quality of life, and reproductive goals.^(1-2,8-13)^ In superficial peritoneal endometriosis, laparoscopic excision or ablation may be performed. Available randomized trials and systematic reviews show heterogeneous results, with no consistent differences in short-term pain relief between techniques and uncertain long-term benefits regarding recurrence or quality of life.^(1,2,9,14)^ In women with mild symptoms or incidental disease, surgery is unlikely to provide additional benefit over optimized medical management. For ovarian endometriomas, laparoscopic cystectomy by excision is preferred over drainage or ablation, as it is associated with lower rates of pain recurrence, endometrioma recurrence, and need for repeat surgery.^(1,9,15,16)^ However, excisional surgery is consistently associated with a postoperative decline in ovarian reserve, with meta-analyses reporting reductions in anti-Müllerian hormone levels of approximately 30–40 percent, particularly after bilateral or repeat procedures.^(2,15,16)^ Fertility outcomes appear comparable between excisional and ablative approaches, reinforcing the importance of individualized counseling and fertility-preserving surgical techniques in women desiring pregnancy.^(2,10,16,17)^ Deep infiltrating endometriosis requires advanced surgical expertise and careful multidisciplinary planning, especially when bowel, bladder, ureteral, or extra-pelvic involvement is present.^(1,5,8,12,18)^ Laparoscopic excision of deep lesions is associated with meaningful improvements in pain, quality of life, and organ-related symptoms in women with severe or refractory disease.^(1,5)^ These benefits must be balanced against higher perioperative risks and potential long-term morbidity, underscoring the importance of referral to specialized centers and tailored surgical extent based on symptom severity and patient priorities. Across all phenotypes, patient-centered factors are central to surgical decision making. Symptom burden, quality of life impairment, reproductive goals, and patient-reported outcomes should guide indications and surgical planning.^(8,10,12,13)^ Postoperative hormonal therapy is recommended for most women not seeking immediate pregnancy, as it may reduce recurrence and prolongs symptom-free intervals, regardless of disease phenotype.^(6,8,13)^ Despite increasing evidence, important gaps remain, particularly regarding long-term comparative outcomes by phenotype, fertility preservation strategies, and repeat surgery. Surgical decisions should therefore remain individualized, weighing expected benefits against risks of recurrence, complications, and diminished ovarian function. These differences in benefit–risk balance across disease phenotypes are summarized in chart 1.

**Chart 1:** Surgical strategies according to endometriosis phenotype

Disease phenotype	Preferred surgical approach	Expected benefits	Main risks and limitations
Superficial peritoneal endometriosis	Laparoscopic excision or ablation	Modest and variable improvement in pain and quality of life in selected symptomatic women	Limited and heterogeneous evidence; no clear superiority between techniques; benefit uncertain in minimally symptomatic disease
Ovarian endometrioma	Laparoscopic cystectomy by excision, with ovarian-sparing technique	Lower rates of pain recurrence and cyst recurrence; possible improvement in spontaneous pregnancy in selected cases	Clinically relevant reduction in ovarian reserve, particularly after bilateral or repeat surgery; benefit on fertility not universal
Deep infiltrating endometriosis	Laparoscopic excision in specialized multidisciplinary centers	Significant improvement in pain, quality of life, and organ-related symptoms when appropriately indicated	Higher surgical complexity and risk; potential long-term functional sequelae; not indicated in asymptomatic disease without organ dysfunction

### How should ovarian endometriomas be surgically managed to minimize damage to ovarian reserve?

Surgical management of ovarian endometriomas should be individualized to minimize damage to ovarian reserve, integrating symptom burden, disease characteristics, imaging findings, quality of life impact, and reproductive goals. Surgery may be indicated in women with persistent or severe pain, large cysts generally exceeding 5 cm, suspicious imaging features, infertility in selected cases, or failure of medical management. However, decision making must recognize that both the presence of an endometrioma and surgical intervention itself can negatively affect ovarian reserve, particularly in women with bilateral disease or prior ovarian surgery.^(1,2,15,19-22)^ Excisional surgery by laparoscopic cystectomy remains the most effective approach for pain relief and reduction of endometrioma recurrence. Pooled analyses consistently demonstrate a postoperative decline in anti-Müllerian hormone levels of approximately 30–40 percent after cystectomy, with greater impact following bilateral or repeat procedures.^(13,15,23)^ Although antral follicle count may partially recover over time, this does not necessarily reflect full functional ovarian reserve. Ablative techniques, including laser or plasma energy, are associated with a smaller decline in anti-Müllerian hormone and may better preserve ovarian tissue, but are linked to higher recurrence rates.^(16,20,24)^ These approaches may be considered in women with diminished ovarian reserve, bilateral endometriomas, previous ovarian surgery, or strong fertility preservation priorities, where minimizing ovarian damage outweighs the risk of recurrence. Hemostasis technique is a critical determinant of ovarian preservation. Bipolar coagulation is consistently associated with greater thermal damage and should be avoided whenever possible. When necessary, the use of electrosurgery should be minimized, and alternative methods, such as suturing, laser coagulation, or plasma energy, are preferred to limit injury to healthy ovarian tissue.^(16,20,25)^ Expectant management represents a reasonable option for asymptomatic women or those primarily pursuing fertility, as it avoids additional surgical compromise of ovarian reserve and facilitates earlier access to assisted reproductive technologies. This strategy requires careful counseling regarding the potential for cyst progression, symptom development, and technical challenges during oocyte retrieval.^(15,21,24)^ Medical therapy may improve pain but does not eliminate endometriomas and should not be considered definitive treatment for cyst resolution. Preoperative counseling is essential and should include explicit discussion of the impact of both disease and surgery on ovarian reserve, realistic fertility expectations, and available fertility preservation strategies, such as oocyte or embryo cryopreservation. Assessment of ovarian reserve using anti-Müllerian hormone and antral follicle count is recommended before surgery, particularly in women with bilateral disease, prior ovarian surgery, or reproductive plans.^(2,15,23)^ Multidisciplinary planning may further optimize outcomes in complex cases. Surgical management of ovarian endometriomas should therefore balance effective symptom control and recurrence reduction against the risk of ovarian reserve impairment. Cystectomy remains the preferred approach for most women with significant symptoms, while ablative or conservative strategies may be appropriate in carefully selected patients with high reproductive priority. Avoidance of bipolar coagulation, thorough preoperative counseling, and individualized planning are essential components of optimal care.

### Is excision superior to ablation for the surgical treatment of endometriosis in all clinical scenarios?

Excision is not universally superior to ablation across all forms of endometriosis. The relative effectiveness of each technique depends on disease phenotype, symptom burden, anatomic extent, and patient priorities. For ovarian endometriomas, excisional surgery by cystectomy provides superior long-term outcomes in terms of pain relief, reduction of cyst recurrence, and lower need for repeat surgery when compared with ablative techniques.^(1,2,16,25,26)^ These benefits are consistently supported by randomized trials and systematic reviews. However, excision is associated with a clinically relevant reduction in ovarian reserve, particularly reflected by postoperative declines in anti-Müllerian hormone, with greater impact after bilateral procedures or repeat surgery. As spontaneous pregnancy rates appear similar between excision and ablation during the first postoperative year, surgical planning must balance symptom control and recurrence prevention against the risk of ovarian function impairment, especially in women with reproductive goals.^(2,13,16,25,26)^ In superficial peritoneal endometriosis, available evidence does not demonstrate a clear advantage of excision over ablation. Randomized controlled trials and meta-analyses show comparable outcomes for pain relief, quality of life improvement, and recurrence at medium- and long-term follow-up.^(14,27-29)^ In this setting, surgical choice should be individualized, guided by symptom severity, distribution of disease, surgeon expertise, and patient expectations. Conservative surgical strategies aimed at symptom relief rather than complete eradication are appropriate, and surgery may not be necessary for women with minimal symptoms.^(9,14,28,29)^ In deep infiltrating endometriosis, excision remains the preferred approach, particularly when lesions involve the bowel, bladder, ureter, or other critical structures. Complete excision is associated with significant improvement in pain, functional outcomes, and quality of life, as well as lower recurrence rates compared with incomplete or non-excisional approaches.^(1,8,13)^ These procedures are technically demanding and associated with higher perioperative risk, reinforcing the need for experienced surgeons and multidisciplinary management in specialized centers. Preoperative counseling is essential to balance expected benefits against the risk of complications and long-term functional sequelae. Across all phenotypes, surgical strategy should be tailored rather than uniform. Disease type, symptom burden, reproductive plans, and patient priorities should guide decision making. Long-term fertility outcomes and ovarian reserve preservation remain incompletely defined, underscoring the need for further high-quality, phenotype-specific comparative studies.

### What is the role of surgery in deep endometriosis, particularly in women without significant pain symptoms?

Deep infiltrating endometriosis is defined by lesions penetrating more than 5 mm beneath the peritoneal surface and frequently involves the bowel, bladder, ureter, or other pelvic structures. Although commonly associated with severe pain, a subset of women may be asymptomatic or report minimal symptoms. Despite limited pain, these patients may still harbor lesions capable of causing silent organ compromise, including ureteral obstruction with hydronephrosis or progressive bowel involvement.^(18,30,31)^ Surgical treatment for deep infiltrating endometriosis should primarily be reserved for women with clinically significant pain, documented organ dysfunction, failure, impossibility or intolerance of hormonal medical therapy, or when malignancy cannot be excluded. In women without relevant pain symptoms, surgery is generally indicated only in the presence of objective evidence of organ compromise, such as silent hydronephrosis, bowel stenosis, progressive loss of organ function, or indeterminate imaging findings raising concern for malignancy.^(1,30,32)^ Routine excision of deep lesions in asymptomatic women is not recommended, as the balance between benefit and harm is unfavorable in the absence of symptoms or functional impairment. Surgery for deep endometriosis carries substantial risks, which increase with lesion depth and organ involvement. Procedures requiring bowel resection, ureterolysis, or reconstruction are associated with higher rates of perioperative complications and potential long-term functional sequelae.^(18,30)^ Moreover, surgery is not curative; recurrence is common, and postoperative medical therapy is often required to reduce symptom relapse and disease progression.^(1,2,8,14)^ While surgery offers clear benefits for symptomatic women, these advantages have not been demonstrated in asymptomatic patients, in whom potential harm may outweigh uncertain benefit.^(1,2,30,32)^ In the absence of significant pain or organ dysfunction, hormonal medical therapy represents the preferred first-line approach. Progestins and combined estrogen–progestin therapies provide effective symptom control in most patients and can be combined with structured clinical and imaging surveillance.^(2,3,9,32)^ Surgery should be reserved for patients who develop symptoms, demonstrate objective progression, or present new evidence of organ compromise. Shared decision making is essential and should include discussion of disease natural history, therapeutic alternatives, surgical risks, and patient priorities.^(2,8,10,32)^ Current evidence does not support prophylactic surgery for asymptomatic deep infiltrating endometriosis. Management should therefore remain individualized, guided by objective findings rather than anatomical extent alone, and aligned with patient values and long-term goals.

### How should bowel and urinary tract involvement be managed surgically?

Surgical management of bowel and urinary tract involvement in endometriosis should be selective and individualized, reserved for women with refractory symptoms, documented organ dysfunction, risk of silent compromise, or suspicion of malignancy. Decision making must integrate disease phenotype, symptom severity, quality of life impact, reproductive goals, and surgical risk, and should occur within a multidisciplinary framework in experienced centers.^(1,8,13,26)^ Accurate preoperative assessment is essential and relies on detailed clinical evaluation combined with advanced imaging, particularly transvaginal ultrasound and magnetic resonance imaging, to define lesion depth, extent, and relationship with adjacent organs. Structured disease mapping informs surgical planning, choice of technique, and the need for colorectal or urologic involvement.^(1,13,33)^ In bowel endometriosis, surgical strategy depends on lesion depth, circumference involvement, and the presence of stenosis or obstruction. Superficial or limited muscular lesions are preferentially managed with shaving excision, which is associated with lower complication rates and preservation of bowel function. Disc excision or segmental resection should be reserved for transmural disease, multifocal lesions, or cases causing significant luminal narrowing or obstruction.^(13,33-35)^ Whenever feasible, nerve-sparing techniques should be employed to reduce postoperative bowel, bladder, and sexual dysfunction. Minimally invasive approaches, including laparoscopy and robotic surgery, are recommended in specialized centers, as they allow precise dissection while minimizing morbidity.^(33-35)^ Urinary tract endometriosis requires particular caution due to the risk of silent progression, especially in ureteral disease. Bladder involvement is usually treated with partial cystectomy, aiming for complete excision of infiltrating lesions while preserving bladder capacity and function. Intraoperative cystoscopy and careful ureteral identification enhance surgical safety.^(2,26)^ Extrinsic ureteral disease may be managed with ureterolysis, whereas intrinsic involvement or significant distal obstruction often requires ureteroneocystostomy. In cases of severe hydronephrosis, preoperative ureteral stenting or nephrostomy may be necessary to protect renal function. Close collaboration with urology is essential to optimize outcomes and minimize complications.^(2,26)^ Surgery is indicated for women with severe pain refractory to medical therapy, bowel obstruction, hydronephrosis, recurrent urinary infections, or progressive loss of organ function. In asymptomatic women, surgery is generally not recommended, except in the presence of silent ureteral obstruction or objective evidence of progressive bowel compromise, as surgical risks may outweigh potential benefits.^(2,13,26)^ Hormonal therapy remains first-line treatment for symptom control and may be continued postoperatively to reduce recurrence. However, medical therapy does not reverse established anatomic obstruction and should not delay surgery when organ function is threatened.^(1,2,13)^ Overall, bowel and urinary tract involvement should be managed using a phenotype-driven, organ-sparing, and multidisciplinary approach. Surgical intervention should aim to relieve symptoms, preserve organ function, and minimize long-term morbidity, with postoperative medical therapy and structured follow-up as integral components of care.^(1,2,8,13,26)^

### What are the risks and limited benefits of repeat surgery for endometriosis?

Repeat surgical treatment for endometriosis in women with persistent or recurrent symptoms after prior surgery is associated with increased risk and limited long-term benefit. Each subsequent operation carries a higher likelihood of intraoperative complications, including bleeding, infection, adhesion formation, and injury to pelvic organs, as well as a cumulative risk of ovarian reserve loss, particularly after repeated excision of ovarian endometriomas.^(36,37)^ Surgical complexity increases with each reintervention due to distorted anatomy and dense adhesions, resulting in longer operative times and a greater probability of organ injury.^(37)^ These risks are particularly relevant in women of reproductive age, in whom repeated ovarian surgery may further compromise fertility potential. Symptom recurrence after repeat conservative surgery remains common. Reported cumulative rates of pain recurrence range from 20% to 40%, and approximately 15% to 20% of women require additional surgery over long-term follow-up.^(38-40)^ While some patients experience temporary symptom improvement, sustained pain relief and durable quality-of-life benefits are inconsistent, especially in women with centralized pain mechanisms, extensive disease, or prior hysterectomy.^(2,41)^ Importantly, repeat surgery does not reliably improve fertility outcomes and may further impair reproductive potential through cumulative damage to ovarian tissue and pelvic anatomy.^(2,41)^ Current evidence does not support a strategy of repetitive surgical intervention for symptom control in endometriosis. Contemporary international recommendations emphasize long-term postoperative hormonal suppression as the main strategy to reduce recurrence and control symptoms after initial surgery. For most women with persistent or recurrent symptoms, optimized medical management and multidisciplinary care are preferred over repeat surgical intervention.^(6,13,42)^ Repeat surgery should therefore be reserved for carefully selected cases, such as those with progressive organ dysfunction, suspicion of malignancy, or severe symptoms refractory to optimized medical therapy. In all situations, shared decision making is essential, with explicit counseling regarding surgical risks, limited expected benefits, alternative treatment options, and the chronic nature of the disease. Repeat surgery for endometriosis should be approached with caution and limited to well-defined indications. For most women, a shift toward long-term medical management and coordinated multidisciplinary care offers a more favorable balance between benefit and harm.

### Should endometriosis surgery be considered a definitive treatment or part of a long-term management strategy?

Endometriosis surgery should not be considered a definitive treatment. Current evidence consistently demonstrates that endometriosis is a chronic, systemic disease, and that surgical intervention represents one component of a broader, long-term management strategy rather than a curative approach.^(1,2,8)^ Although surgery plays a central role in selected clinical scenarios, including refractory pain, organ dysfunction, infertility in carefully selected cases, or suspicion of malignancy, recurrence after surgery is common. Long-term follow-up studies indicate that up to 40–45% of women experience recurrence of pain, and a substantial proportion require additional treatment or repeat surgery within five to seven years.^(1,8,41)^ These data highlight the limitations of surgery when used as a standalone intervention. International guidelines emphasize that the primary objectives of surgery are symptom relief, restoration of anatomy when necessary, and management of complications, rather than eradication of disease.^(2,13)^ The ESHERE explicitly recommends that surgery be integrated into an individualized care plan and followed by appropriate postoperative medical therapy in women not seeking immediate pregnancy, in order to reduce recurrence and prolong symptom-free intervals.^(13)^ Postoperative hormonal suppression is a key element of long-term management. Continuous use of estrogen–progestin combinations or progestins significantly reduces the risk of pain recurrence and repeat surgery when compared with surgery alone.^(6,43)^ This supports the concept that disease activity persists beyond surgical excision, reflecting inflammatory, hormonal, and neurogenic mechanisms that are not fully addressed by surgery. Treatment decisions should therefore incorporate disease phenotype, symptom burden, impact on quality of life, and reproductive goals. Surgical morbidity, cumulative adhesion formation, and the risk of diminished ovarian reserve, particularly following ovarian surgery, further support a cautious and strategic use of surgical intervention.^(1,8,10)^Multidisciplinary care and shared decision making are essential to align treatment choices with patient priorities and long-term outcomes. Endometriosis surgery should be regarded as part of a long-term, multidisciplinary management strategy rather than a definitive treatment. Optimal care requires integration of surgery with sustained medical therapy, ongoing monitoring, and individualized planning based on disease characteristics and patient goals, in accordance with contemporary international guidelines.

### What is the role of postoperative hormonal therapy in symptom control and prevention of disease recurrence?

Postoperative hormonal therapy plays a central role in the long-term management of endometriosis following surgery and should be regarded as a continuation of disease control rather than an optional adjunct. Robust evidence consistently demonstrates that surgery alone is insufficient to suppress disease activity and that, in the absence of postoperative medical therapy, recurrence of symptoms and lesions is common across all major endometriosis phenotypes. Endometriosis is a chronic, estrogen-dependent inflammatory disease, and surgical excision does not eliminate the systemic, hormonal, and neurogenic mechanisms that sustain disease activity.^(1,2,6)^ High-quality evidence from systematic reviews and meta-analyses shows that women receiving postoperative hormonal suppression have a substantially lower risk of pain recurrence and repeat surgery compared with those managed expectantly after surgery. Risk reductions of approximately 50–60% have been consistently reported, regardless of disease phenotype, including superficial peritoneal disease, ovarian endometriomas, and deep infiltrating endometriosis.^(3,6,44)^ These findings support the concept that residual microscopic disease and ongoing inflammatory pathways persist after surgical excision and require long-term suppression. International guidelines strongly endorse postoperative hormonal therapy for women not seeking immediate pregnancy. The ESHERE explicitly recommends postoperative medical treatment to reduce recurrence of pain and lesions, emphasizing that therapy should be continued long term, as discontinuation is associated with rapid symptom relapse.^(13)^ Similar positions are reflected in other contemporary reviews and consensus statements, which highlight the importance of individualized agent selection based on tolerability, comorbidities, and reproductive plans.^(1,2)^ First-line postoperative options include combined estrogen–progestin contraceptives, oral or injectable progestins, and the levonorgestrel-releasing intrauterine system. Continuous regimens are consistently superior to cyclic use for pain control and recurrence prevention.^(1,3)^ In women with ovarian endometriomas, long-term progestin therapy, particularly dienogest, and LNG-IUS placement after cystectomy are associated with the lowest recurrence rates and reduced need for repeat surgery.^(7,45)^ For deep infiltrating endometriosis, continuous oral contraceptives and progestins are effective for maintaining symptom control, although comparative data between specific agents remain limited.^(3,13)^ GnRH agonists and antagonists are effective in suppressing disease activity but are generally reserved for second-line use due to hypoestrogenic adverse effects, cost, and concerns regarding bone health. When used beyond short durations, add-back therapy is mandatory to improve tolerability and allow extended treatment.^(2,44)^ Duration of therapy is a critical determinant of success. Postoperative hormonal suppression should be maintained until pregnancy is desired or menopause occurs, as discontinuation is consistently associated with high rates of symptom recurrence.^(3,13)^ In women with immediate fertility goals, postoperative hormonal therapy is contraindicated, and alternative strategies should be pursued.

### How should postoperative management differ between women seeking pregnancy and those not seeking pregnancy?

Postoperative management of endometriosis must be fundamentally guided by reproductive intentions, as treatment strategies that effectively suppress disease activity are inherently incompatible with conception. Given the chronic and estrogen-dependent nature of endometriosis, long-term symptom control and prevention of disease recurrence rely on sustained postoperative medical therapy in most women who are not actively seeking pregnancy, as previously explained.^(1,4,6,9)^ For women not seeking pregnancy, immediate initiation of postoperative hormonal therapy is strongly recommended. High-quality evidence demonstrates that hormonal suppression after surgery reduces the risk of symptom recurrence and disease relapse by approximately 50–60% compared with expectant management.^(1,6,45)^ Continuous combined oral contraceptives, progestins, dienogest, and the levonorgestrel-releasing intrauterine system represent first-line options, with continuous regimens consistently outperforming cyclic schedules for pain control and recurrence prevention.^(3,4,9,44,45)^ ESHRE guidelines explicitly recommend long-term postoperative hormonal therapy in this population, emphasizing that interruption of treatment is associated with rapid symptom relapse and renewed disease activity.^(13,46)^ In women with ovarian endometriomas, dienogest and LNG-IUS appear particularly effective in reducing postoperative recurrence following cystectomy.^(45)^ Because the protective effect of hormonal therapy is rapidly lost after discontinuation, treatment should generally be maintained until pregnancy is desired or menopause is reached.^(3,4,45)^ In contrast, postoperative hormonal therapy is contraindicated in women actively seeking pregnancy, as ovulation suppression delays conception.^(1,9,47)^ In this group, postoperative management is expectant, with close clinical and imaging follow-up to monitor for symptom recurrence or disease progression. The increased risk of recurrence during this period is generally considered acceptable when the probability of conception is reasonable.^(47)^ Short-term postoperative use of GnRH agonists has been proposed in selected infertile women to improve subsequent fertility; however, evidence remains inconsistent, and this approach is not routinely recommended, as it delays attempts at conception without clear universal benefit.^(48,49)^ For women undergoing assisted reproductive technologies, available data are largely reassuring, although individualized counseling and multidisciplinary coordination remain essential.^(47)^ Postoperative management should be primarily guided by reproductive intention, with distinct strategies for women seeking pregnancy and those not pursuing conception, as summarized in chart 2.

**Chart 2:** Postoperative management according to reproductive intention

Reproductive intention	Recommended postoperative strategy	Rationale
Not seeking pregnancy	Long-term continuous hormonal suppression	Reduces symptom recurrence and disease relapse; surgery alone is insufficient for long-term control
Seeking natural conception	Expectant management with close follow-up	Hormonal suppression delays ovulation; recurrence risk accepted when probability of conception is reasonable
Infertility or low probability of natural conception	Early referral to assisted reproductive technologies	Surgery does not replace ART and may further compromise ovarian reserve
Transitioning reproductive goals	Periodic reassessment and individualized adjustment	Endometriosis management must adapt to changing life priorities

### Is long-term hormonal maintenance recommended after successful surgical treatment of endometriosis?

Long-term hormonal maintenance therapy is recommended after successful surgical treatment of endometriosis in women not actively seeking pregnancy, reflecting the chronic and estrogen-dependent nature of the disease. Without postoperative suppression, recurrence of pain or lesions occurs in up to 40–45% of women within two to five years after surgery, and a substantial proportion require repeat interventions.^(1,3,4,9,50)^ Multiple systematic reviews and network meta-analyses demonstrate that long-term hormonal maintenance reduces recurrence risk by more than 50% compared with expectant management and provides superior long-term pain control.^(6,7,45)^ These benefits are observed across different disease phenotypes, including ovarian and deep infiltrating disease. ESHRE guidelines strongly recommend postoperative hormonal maintenance for women not desiring pregnancy and emphasize that therapy should be continued long term, as discontinuation is associated with rapid symptom relapse.^(13)^ First-line maintenance options include combined estrogen–progestin contraceptives, oral or depot progestins, and the levonorgestrel-releasing intrauterine system.^(1,3,4,9,50-52)^ In ovarian endometriosis, dienogest and LNG-IUS show the most robust evidence for preventing endometrioma recurrence after cystectomy and are frequently preferred when ovarian reserve preservation and long-term tolerability are priorities.^(7,45,53)^ In deep infiltrating disease, long-term progestin-based maintenance is also supported, although comparative data remain limited.^(3,4,50,52)^ Hormonal maintenance should be continued until pregnancy is desired or menopause occurs. The protective effect of therapy is rapidly lost after discontinuation, reinforcing the need for sustained treatment in women not seeking conception.^(3,4,50)^ In women actively seeking pregnancy, maintenance therapy is contraindicated, and expectant management with close surveillance is recommended.

### Which outcomes should be used to evaluate the success of endometriosis surgery beyond short-term pain relief?

Assessment of surgical success in endometriosis must extend beyond short-term pain relief and reflect the chronic, recurrent, and systemic nature of the disease. Contemporary management frameworks emphasize that immediate postoperative symptom improvement is an incomplete and potentially misleading indicator of long-term benefit. Instead, surgical outcomes should be evaluated using longitudinal, patient-centered, and phenotype-specific measures that capture durability of symptom control, functional recovery, and alignment with individual treatment goals.^(8,41,54)^ Sustained symptom control over time represents a core outcome. Although many women experience early postoperative improvement, long-term data demonstrate that pain recurrence remains frequent, with up to 40–45% of patients reporting recurrent symptoms within several years after surgery, and a substantial proportion requiring further interventions.^(8,41,54)^ Durable reduction in dysmenorrhea, dyspareunia, and non-cyclic pelvic pain over extended follow-up therefore provides a more meaningful measure of surgical effectiveness than early postoperative outcomes alone. Recurrence-related outcomes constitute another critical domain. These include symptom relapse, need for repeat surgery, and objective disease progression on imaging when available. Such outcomes reflect not only the completeness and appropriateness of the initial surgical approach but also the adequacy of postoperative management, particularly the use of long-term hormonal maintenance strategies.^(41,54)^ High reintervention rates despite technically successful surgery underscore the limitations of surgery as a standalone treatment in a chronic disease context. Health-related quality of life is increasingly recognized as a central endpoint and should be systematically assessed using validated patient-reported outcome measures, such as the Endometriosis Health Profile (EHP-30 or EHP-5) or generic instruments like the SF-36. Long-term studies consistently show improvements in physical, emotional, sexual, and social domains following surgery, particularly in women with deep infiltrating disease; however, trajectories are heterogeneous, and a subset of patients experience persistent or recurrent impairment despite adequate surgical management.^(55-57)^ Fertility and reproductive outcomes are essential in women with reproductive goals and must be interpreted cautiously. Relevant endpoints include spontaneous conception rates, time to pregnancy, need for assisted reproductive technologies, and preservation of ovarian reserve. While surgery may improve spontaneous pregnancy rates in selected phenotypes, excision of ovarian endometriomas is associated with a measurable reduction in ovarian reserve, highlighting the need to balance potential reproductive benefit against long-term ovarian function.^(1,2,14,54)^ Phenotype-specific outcomes should be explicitly incorporated into evaluation frameworks. Deep infiltrating endometriosis and associated conditions, such as adenomyosis, are often associated with greater improvements in pain and quality of life, but also carry higher risks of complications, recurrence, and reintervention. Surgical success must therefore be interpreted in the context of baseline disease severity and anatomic distribution.^(1,54,56)^ Finally, patient-reported satisfaction and goal attainment are increasingly recognized as essential indicators of success. Alignment between surgical outcomes and individual patient priorities—such as pain control, fertility preservation, daily functioning, or avoidance of repeat surgery—should be explicitly assessed and documented. Taken together, evaluation of endometriosis surgery should rely on long-term symptom control, recurrence-related outcomes, quality-of-life measures, reproductive outcomes, phenotype-specific risks and benefits, and patient-reported satisfaction, rather than short-term pain relief alone. This multidimensional approach is consistent with contemporary international guidance and supports a realistic, patient-centered assessment of surgical effectiveness in a chronic disease setting.

### How should shared decision making be structured throughout surgical treatment and long-term follow-up

**Figure 2:**
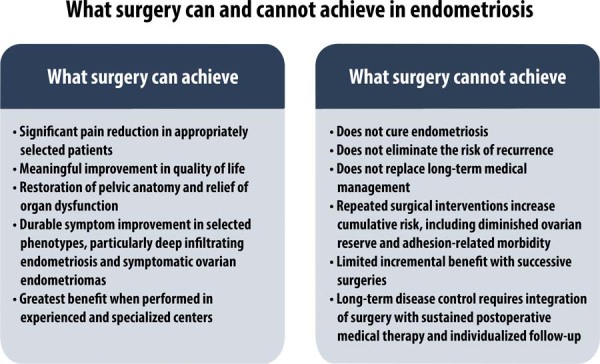
Realistic benefits and inherent limitations of surgical treatment in endometriosis

Shared decision making in endometriosis care should be structured as a continuous, dynamic, and patient-centered process that extends from initial assessment through surgical planning and long-term follow-up. This approach reflects the chronic and heterogeneous nature of endometriosis and acknowledges that treatment goals, symptom burden, and reproductive priorities frequently evolve over time.^(1,12,58)^ The process begins at the first specialist consultation with transparent communication regarding the natural history of endometriosis, the variability of symptom expression, and the realistic objectives of treatment. Patients should be informed that surgery is not curative and that symptom persistence or recurrence is common, even after technically successful procedures.^(1,58)^ Baseline evaluation should include detailed characterization of pain, multisystem symptoms, bowel and urinary involvement, and reproductive history, complemented by validated patient-reported outcome measures to capture quality-of-life impact.^(12,59-61)^ Treatment planning should integrate medical, surgical, and combined strategies, with explicit discussion of benefits, limitations, and risks associated with each option. This includes recurrence rates, potential impact on ovarian reserve, perioperative morbidity, and the role of long-term hormonal maintenance.^(2,17,43,58,62,63)^ In complex disease phenotypes, particularly deep infiltrating disease or cases involving fertility considerations, a multidisciplinary model involving pain specialists, fertility experts, colorectal or urologic surgeons, and mental health professionals is recommended to support balanced and informed decisions.^(43,58,62,63)^ Decisions regarding surgery should be individualized according to disease phenotype, symptom severity, and reproductive goals. Counseling must address the spectrum from conservative to more extensive procedures, fertility preservation strategies, and the timing of surgery relative to assisted reproductive technologies when relevant.^(2,17,59)^ Clear discussion of surgical risks, including diminished ovarian reserve, organ injury, and the possibility of repeat interventions, is essential to align expectations and minimize decisional regret.^(60,61)^ Shared decision making continues after surgery through structured long-term follow-up. Management plans should be revisited regularly to address symptom recurrence, changes in quality of life, and evolving fertility intentions.^(1,12,43,62)^ For women not seeking pregnancy, postoperative hormonal maintenance should be discussed as a strategy to reduce recurrence and prolong symptom control. For those pursuing pregnancy, counseling should focus on expectant management, monitoring strategies, and fertility planning. Repeated assessment using patient-reported outcome measures facilitates adaptive, goal-oriented care over time.^(43,60,61)^ An effective shared decision-making framework prioritizes patient values, preferences, and lived experience. Clinicians should openly acknowledge uncertainties in the evidence base, particularly regarding long-term outcomes and optimal follow-up strategies, and address disparities in access to specialized care and continuity of follow-up.^(12,59)^ Shared decision making in endometriosis surgery should therefore be viewed as an ongoing, multidisciplinary, and individualized process that integrates symptom burden, disease phenotype, quality of life, and reproductive goals from initial assessment through long-term follow-up. This model aligns with contemporary international guidance and supports treatment strategies that remain responsive to patient priorities over time. To align expectations and reinforce realistic treatment goals, the key capabilities and limitations of surgery in endometriosis are summarized in figure 2.

## Final considerations

Surgical treatment of endometriosis must be individualized and guided by disease phenotype, symptom burden, reproductive goals, and patient priorities. While surgery plays an important role in selected clinical scenarios particularly in deep infiltrating disease and symptomatic ovarian endometriomas it should not be applied indiscriminately or viewed as a definitive solution. Long-term success depends on integration of appropriate surgical technique, postoperative medical therapy, structured perioperative care, and ongoing multidisciplinary follow-up. A personalized, evidence-informed approach remains essential to optimize outcomes, preserve fertility when relevant, and minimize long-term harm in women living with endometriosis.
